# Strength Training Habits in Amateur Endurance Runners in Spain: Influence of Athletic Level

**DOI:** 10.3390/ijerph17218184

**Published:** 2020-11-05

**Authors:** Felipe García-Pinillos, Carlos Lago-Fuentes, Diego Jaén-Carrillo, Pascual Bujalance-Moreno, Pedro Ángel Latorre-Román, Luis Enrique Roche-Seruendo, Rodrigo Ramirez-Campillo

**Affiliations:** 1 Department of Sports and Physical Education, University of Granada, 18071 Granada, Spain; fgpinillos@ugr.es; 2 Department of Physical Education, Sport and Recreation, Universidad de La Frontera, Temuco, Chile; 3 Faculty of Education and Sport Sciences, University of Vigo, 36310 Pontevedra, Spain; carloslagofuentes@hotmail.com; 4 Faculty of Health Sciences, European University of Atlantic, 39011 Santander, Spain; 5 Faculty of Health Sciences, Universidad San Jorge, 50830 Villanueva de Gállego, Zaragoza, Spain; leroche@usj.es; 6 Department of Corporal Expression, Campus de Las Lagunillas, University of Jaen, 23071 Jaen, Spain; pascualbujalancemoreno@gmail.com (P.B.-M.); platorre@ujaen.es (P.Á.L.-R.); 7 Laboratory of Human Performance, Quality of Life and Wellness Research Group, Department of Physical Activity Sciences, Universidad de Los Lagos, Osorno, Chile; r.ramirez@ulagos.cl

**Keywords:** long-distance athletes, resistance training, plyometric exercise, running, human physical conditioning

## Abstract

This study determined the strength training (ST) habits of amateur endurance runners in Spain regarding athletic level. A sixteen-item online questionnaire comprised of (i) demographic information, (ii) performance, and (iii) training contents was completed by 1179 athletes. Five group levels were determined according to the personal best times of the athletes in a 10-km trial (LG1: level group 1, 50–55 min; LG2: level group 2, 45–50 min; LG3: level group 3, 40–45 min; LG4: level group 4, 35–40 min; LG5: level group 5, 30–35 min). Most athletes (*n* = 735, 62.3%) perceived ST as being a key component in their training program. Resistance training (RT) was reported as a ST type used by 63.4% of the athletes, 66.9% reported using bodyweight exercises, 46.8% reported using plyometric training, 65.6% reported using uphill runs, and 17.8% reported using resisted runs. The prevalence of runners who excluded ST from their training programs decreased as the athletic performance level increased (18.2% in lower-level athletes vs. 3.0% in higher-level), while the inclusion of RT, bodyweight exercises, plyometric training, and uphill and resisted runs was more frequent within higher-level groups. Most athletes included ST using low-to-moderate loads and high a number of repetitions/sets comprised of RT, plyometric training, resisted runs, and core, respiratory, and foot muscles training.

## 1. Introduction

Running events appear to have become particularly popular in recent years [1]. For instance, from 2001 to 2012, the number of finishers at the 20 largest road races rose from 866,000 to 1,594,000 [2]. However, running is not a risk-free sport. A recent meta-analysis has highlighted that, depending on the type of runner, injury definition, and length of follow-up, the reported running-related injury (RRI) incidence rate ranges from 2.5 to 33.0 injuries per 1000 h of running [3]. Although inter-study comparisons of injury rates are complicated on account of different methods used and different populations, there is consensus that RRI rates are unacceptably high despite considerable efforts to reduce them [3], particularly in amateur and novice endurance runners [4,5].

Owing to the potentially high risk of sustaining injury [6], its consequences on time-to-recovery and socioeconomic costs [7], and the high number of active runners [2], RRI arguably characterizes an important public health issue. Therefore, injury prevention research approaches and strategies should be prioritized. A recent meta-analysis [8] concluded that programmed physical exercise is effective at reducing the risk of injury and incidence. Specifically, strength training (ST) was shown to be the most effective strategy for preventing sports injuries—more than stretching, proprioception training, or multiple exposure prevention. The authors [8] reported that interventions based on ST might reduce overuse injuries by almost 50%. Therefore, incorporating ST into the training program of endurance runners might be considered as a means in which to reduce the incidence of RRI.

The convenience of ST for endurance runners has been justified not only in terms of minimizing RRI [8] but also in terms of maximizing athletic performance [9,10,11]. In detail, ST focused on neural adaptations has been shown to be one of the most efficient strategies for improving sport performance in athletes [12]. The benefits of ST include improvements in running economy through different mechanisms, such as mechanical efficiency, muscle coordination, and/or motor recruitment patterns [10,11]. Therefore, ST is crucial for endurance runners from two standpoints: maximizing athletic performance and minimizing the risk of injury.

In this context, coaches and athletes should critically evaluate their training programs and ask themselves if they are giving enough importance to ST. Some previous studies have described the training characteristics [13] or the warm-up and cool-down habits [14] of endurance runners. However, the available information about the presence of ST and its characteristics in the training program of endurance runners is limited. A previous study [15] described the year-round training characteristics of 93 athletes who qualified for the 2004 USA Olympic Marathon Trials, and it was identified that elite marathon runners completed more sessions of ST than national-class runners, but with a low frequency (less than once a week for men and twice a week for women). However, Blagrove and colleagues [16] explored the strength and conditioning (S&C) habits of 667 competitive middle- and long-distance runners and found that 23.1% of runners did not include resistance training (RT) or plyometric training in their programs. Both studies produced valuable information on competitive and highly trained endurance runners. However, no consideration was given to amateur or non-competitive endurance runners, therefore excluding a very high proportion of runners.

Although previous studies have described some details about ST habits in highly trained endurance runners, no previous work has focused on the characteristics of training programs in amateur endurance runners and whether their ST workouts differ according to their performance level. Consequently, the purpose of this study was to examine the ST habits of amateur endurance runners in Spain and to determine the influence of potential influencing variables such as athletic level.

## 2. Materials and Methods

### 2.1. Participants

A sample comprised of endurance runners in Spain (*n* = 1179; age range = 18–60 years; age = 35.5 ± 10.8 years) participated in this study. All athletes met the following inclusion criteria: (1) older than 18 years, (2) two or more running sessions per week, (3) able to run 10 km in <55 min. Points 2 and 3 refer to the last 6 months before data collection. After receiving detailed information on the objectives and procedures of the study, each subject signed an informed consent form. Additionally, this study meets the ethical standards of the World Medical Association’s Declaration of Helsinki (2013) and was approved by the Institutional Review Board (Universidad de La Frontera, Temucho, Chile, 005_19).

### 2.2. Procedures

This is a cross-sectional study with descriptive purposes. An ad-hoc questionnaire was designed and provided to endurance runners via an online Google Forms survey (https://drive.google.com/open?id=1yYc4-iDWlYQk1QVt42RDZJ0H_oU4W4B4WWQKBYLvjZc). This research project was conducted according to the European General Data Protection Regulation (EU GDPR; EU 2016/679).

After receiving ethical approval from the institutional review board, a pilot test was conducted with a small sample of athletes (*n* = 40) to evaluate the clarity and content of the online Google Forms survey. All the athletes involved in the pilot test gave feedback that the online questionnaire was appropriate and suitable. Subsequently, several sports clubs, federations, and institutes in Spain were contacted through their managers and asked to publicize the study to their athletes. All of the sport organizations were in line with the current data protection regulations implicating that athletes were informed about the potential use of their personal data for research purposes when they provided such information. Then, athletes who accepted the invitation to participate in the study were given a link to the online questionnaire. According to online informed consent procedures, participants were told of the purpose and details of the study through a participant information sheet. After consenting to participate in the study, participants filled in sixteen items, divided into three main question blocks:

(i) Demographic information (i.e., sex and age).
(ii) Information about athletic performance in the last 6 months, including whether the athletes had a coach (through a yes/no question), whether they were a registered athlete (through a yes/no question), and their personal best in a 10-km trial (through nominal scale including different durations ranging from 50–55 min to <30 min).
(iii) Information about their training programs in the last 6 months, including hours and kilometers per week (through nominal scales, e.g., 2–3 h and 30–35 km), perceived importance of ST (through Likert-type scale, 1–3 rating, in which 1 means “ST is perceived as not important” and 3 means “ST is perceived as very important” in their training programs), ST sessions per week (ranging from 0 to 14 sessions per week), duration (ranging from 0 to 180 min per session), type (through nominal scales, including “no ST sessions”, “ST with external load”, “bodyweight exercises”, “plyometric training”, “hill runs”, and/or “resisted runs”), and timing of ST sessions (through nominal scales, including “no ST sessions”, “just before running exercises, same day”, “just after running exercises, same day”, “same day, but at least 4–5 h between running and ST”, and/or “alternating days to running workouts”), range of repetitions per set performed (ranging from 0 to >20), incorporation of core training (through a yes/no question), foot muscles training (through a yes/no question), respiratory muscles training (through a yes/no question).

In order to highlight the potential influence of the athletic level on the ST habits of this group of amateur endurance runners in Spain, five group levels were determined according to the athletes’ personal best time in a 10-km trial (LG1: 50–55 min in a 10-km trial; LG2: 45–50 min in a 10-km trial; LG3: 40–45 min in a 10-km trial; LG4: 35–40 min in a 10-km trial; LG5: 30–35 min in a 10-km trial).

### 2.3. Statistical Analysis

Descriptive data are presented as means and standard deviations for interval variables and as frequencies and percentages for nominal variables. Descriptive data are provided by means of frequencies and percentages. A chi-squared test was conducted to determine differences between the different group levels. This between-group analysis was conducted independently for male and female runners in order to avoid the potential influence of sex. When significant differences were found, a Bonferroni post-hoc test was conducted. All statistical analyses were performed using the software IBM Statistical Package for Social Sciences Statistics, version 22.0 (IBM Inc., Armonk, NY, USA). Statistical significance was set at *p* < 0.05.

## 3. Results

A group of 951 men (80.7%) and 228 women (19.3%) filled in the questionnaire. Of these respondents, 723 athletes (61.3%) had a coach and 546 (46.3%) were registered athletes.

Concerning general information about training, 26.3% of the participants trained from 2 to 5 h per week, 40.1% trained 5–8 h/week, 22.9% trained 8–12 h/week, and the remaining 10.7% trained more than 12 h/week. In terms of kilometers per week, 42.7% ran ≤40 km, 38.4% ran 41–70 km, 15.6% ran 71–100 km, and the remaining 3.3% ran >100 km/week.

**Figure 1:**
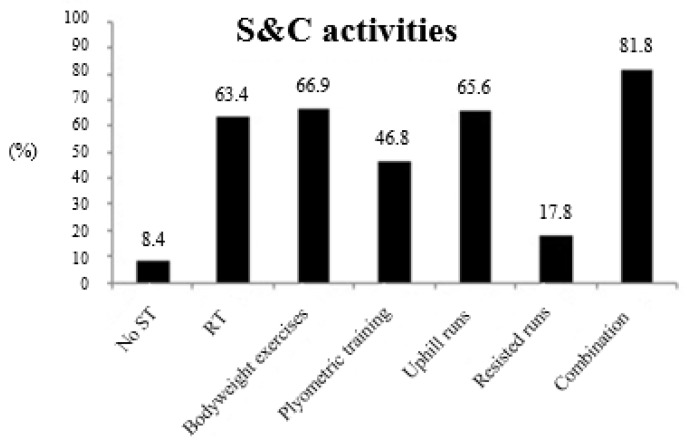
Strength and conditioning (S&C) activities used by endurance athletes (in %). ST: strength training; RT: resistance training.

**Figure 2:**
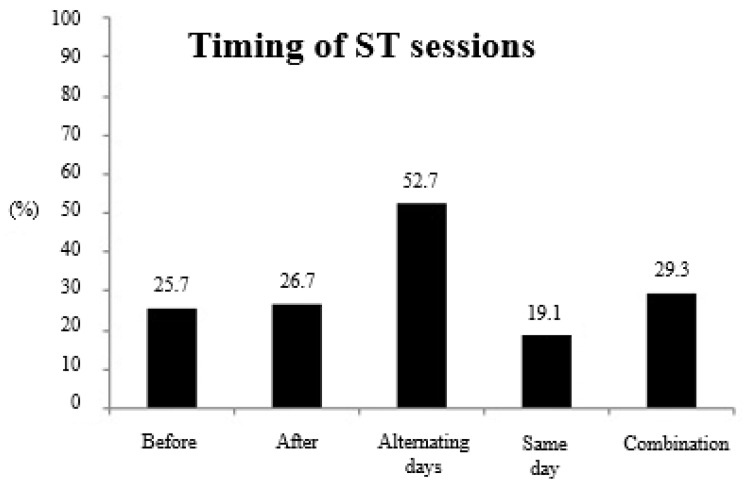
Timing of the strength training (ST) sessions relative to running sessions.

**Figure 3:**
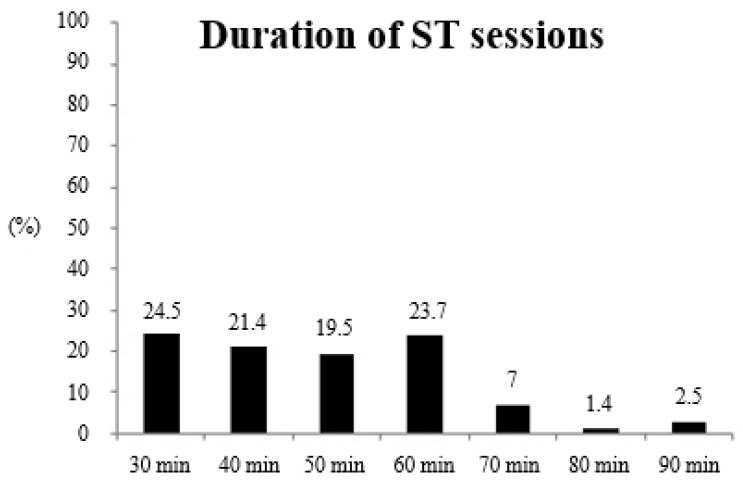
Strength training (ST) volume in terms of duration of the sessions.

**Figure 4:**
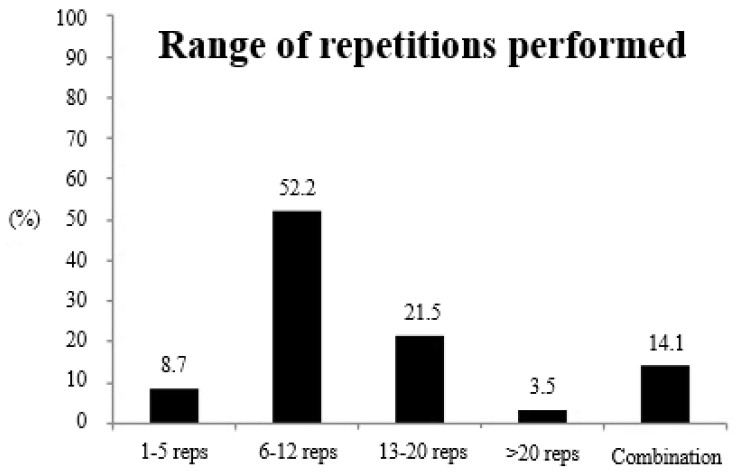
Range of repetitions (reps) used by endurance athletes in their resistance training sessions.

**Figure 5:**
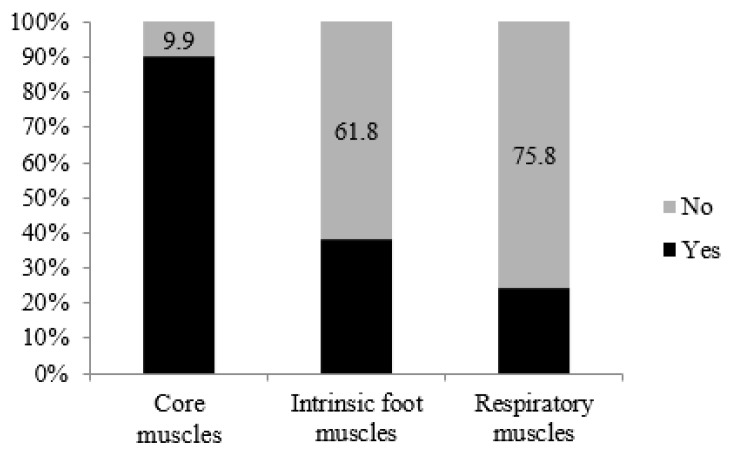
Strength training (ST) habits with special focus on core muscles, foot muscles, and respiratory muscles.

Focusing on the ST habits in endurance runners (Figure 1, Figure 2, Figure 3, Figure 4 and Figure 5), most of the endurance athletes (735; 62.3%) perceived ST as an important part of the training program, while 32.8% considered it as moderately important and only 4.8% considered it as trivial. The endurance runners surveyed averaged 1.75 ± 1.03 ST sessions per week. Figure 1 shows the S&C activities used by endurance athletes (in %). Whereas 99 athletes (8.4%) did not include any type of ST in their training programs, most (*n* = 1080) athletes included some form of ST in their training programs. Of those who included ST in their training programs, 747 athletes (63.4%) included RT, 789 (66.9%) included bodyweight exercises, 552 (46.8%) included plyometric training, 774 (65.6%) included uphill runs, and 210 (17.8%) included resisted runs.

Figure 2 shows information about the timing of the ST sessions relative to running sessions. Of those athletes who included any type of ST in their training programs (*n* = 1080), most of the endurance athletes (52.7%) usually included ST sessions on alternated days with running sessions, while 29.3% of the athletes combined different times for their ST sessions.

Figure 3 provides information about ST volume in terms of the duration of the sessions. The majority (81.4%) of runners included ST sessions shorter than 60 min.

Figure 4 shows information about the range of repetitions used by endurance athletes in their RT sessions. The majority of participants (63.4%; 747 athletes) included RT sessions in their training programs. Of those who included RT sessions, 81 athletes (8.7%) usually performed 1–5 reps per set, 489 (52.2%) performed 6–12 reps, 201 (21.5%) performed 13–20 reps, 33 (3.5%) performed >20 reps, and 132 athletes (14.1%) combined different ranges of repetitions: 3.0% usually performed all ranges, 6.5% performed >6 reps, 3.6% performed 1–12 reps, and 1.0% of athletes performed >12 reps per set.

Figure 5 provides information about ST habits with a special focus on core muscles, foot muscles, and respiratory muscles. The majority (90.1%) of athletes included core training in their training program, 38.2% included foot muscles training, and 24.2% included respiratory muscles training.

Regarding the influence of athletic level, some between-group differences were found in different items included in the current questionnaire for both male and female athletes. Having a coach was more frequently found in higher-level groups than in lower-level groups regardless of sex (*p* < 0.001). Likewise, higher-level groups showed more hours and kilometers per week than lower-level groups in both male and female endurance athletes (*p* < 0.001). As for the ST sessions per week for male endurance runners, higher-level groups included more sessions than lower-level groups (*p* < 0.001). No significant differences between athletic level groups (*p* = 0.187) were found in the perception of those athletes about the importance of ST. In regard to female athletes, no between-group differences were found in ST per week (*p* = 0.536) or perception of the importance of ST (*p* = 0.475).

Table 1 and Table 2 respectively show the ST habits of male and female amateur endurance runners regarding athletic level. As for male runners (Table 1, *n* = 951), the distribution of athletes regarding athletic performance levels was as follows: LG1 72 (7.6%), LG2 126 (13.2%), LG3 237 (24.9%), LG4 324 (34.1%), and LG5 192 (20.2%). The prevalence of runners who did not include ST in their training programs significantly decreased (*p* < 0.001) as the level increased (19.4% in LG1 vs. 3.1% in LG5), while the inclusion of S&C activities such as RT, bodyweight exercises, plyometric training, and uphill and resisted runs were more frequently found in higher level groups (*p* < 0.001). For example, 56.9% of the LG1 included RT, whereas the prevalence in the LG5 was 73.0%, with the Bonferroni post-hoc test showing significant differences between levels (*p* < 0.05). Additionally, Table 1 provides data about the influence of athletic level on the inclusion of core training (*p* = 0.61), foot muscles training (*p* < 0.001), and respiratory muscles training (*p* < 0.001), with the higher level groups reporting greater prevalence of training foot and respiratory muscles (i.e., the post-hoc test revealed differences between each level of group).

As for female runners (Table 2, *n* = 228), the distribution of athletes regarding athletic performance levels was as follows: LG1 60 (26.3%), LG2 69 (30.3%), LG3 72 (31.6%), LG4 18 (7.9%), and LG5 9 (3.9%). Some between-group differences (*p* = 0.010) were found in S&C activities regarding athletic level (e.g., the LG1 showed the highest prevalence of no ST, 20%). Of note, the LG5 reported the highest prevalence in every item, indicating higher frequency for every S&C activity. Regarding the inclusion of core training, significant between-group differences were observed, with higher frequencies and prevalence for the higher-level groups (e.g., 83.3% for the LG1 and 94.4% for the LG4). No significant between-group differences were found in the frequency and prevalence of female runners including intrinsic foot muscles and respiratory muscles training (*p* = 0.837 and 0.200, respectively).

## 4. Discussion

This study aimed to examine the ST habits of amateur endurance runners and to determine the influence of athletic level on the ST habits. A group of 1179 Spanish endurance runners completed an online questionnaire, and the results leave some important findings. The first notable finding, regarding the presence of ST in the plans of these athletes, is that 8.4% of athletes did not include ST in their training programs. Furthermore, 4.8% of the runners perceived ST as being trivial, despite the multiple benefits reported by previous studies [8,9,10,11]. These results are in line with the hypothesis by Blagrove and colleagues [16] who suggested that some training myths and disinformation among runners may lead them toward inefficient and unhealthy training habits. Compared with the high prevalence (91.6%) of ST in the athletes of the current study, previous studies reported that ~50% of runners did not include ST in their training programs [15] and 23.1% did not include RT or plyometric training in their training programs [16]. According to this, a positive trend might be occurring in endurance runners regarding their training practices.

Another major finding is related to the S&C activities used. The results indicated that most of the endurance athletes included uphill runs (65.6%), RT (63.4%), and bodyweight exercises (66.9%) in their training programs, whereas almost half of the runners surveyed included plyometric training (46.8%) and a lower number (17.8%) included resisted runs. Different training activities have been considered by previous studies, which makes comparisons difficult. For instance, the study by Karp [15] did not analyze the S&C activities used—the study just considered the number of ST sessions per week. Nevertheless, some similarities can be found with other previous studies performed with endurance runners. For example, in a study with Irish runners [17], almost 80% of the runners included RT in their training; also, the systematic review by Blagrove et al. [16] reported a prevalence of ~63%. In considering the different S&C activities, plyometric training was also included in both studies [16,17] with similar prevalence levels (i.e., 35.1% and 35.4%, respectively), although these prevalence levels were lower than our findings (i.e., 46.8%). Besides this, it seems clear that the majority of endurance athletes currently include ST in their weekly routines, which is in agreement with the scientific evidence on this topic [8,9,10,11]. It is well known that neural adaptations derived from ST, RT, and plyometric training have been identified as the most efficient strategies for athletic performance improvement [12], therefore, it seems logical to affirm that these strategies induce improvements in mechanical efficiency, muscle coordination, and motor recruitment patterns [10,11]. Regarding this, the findings reported here may suggest that coaches and athletes should critically revise their training programs and strive to achieve a greater incorporation of RT and plyometric training into their training programs.

Given the importance of heavy RT for endurance runners [8,9,10,11], it might be worthwhile for coaches and athletes to describe the characteristics of their RT sessions. On average, the endurance runners surveyed performed ~1.8 ST sessions per week, which is in agreement with data reported by previous studies [15,16]. Additionally, the results obtained showed that just 8.7% of the surveyed runners included RT workouts based on low volume and high load (e.g., 1–5 reps per set), while most of the runners trained with moderate-to-high volumes and moderate-to-low loads (i.e., 52.2% between 6–12 reps, 21.5% between 13–20 reps, and 3.5% >20 reps). It is worth highlighting that the aforementioned studies [15,16] only considered highly trained athletes, therefore excluding the vast amateur population who run habitually. Griffin et al. [17] highlighted that 36.5% of athletes trained twice a week with weight training, but without specifying the range of intensity of this work. For this reason, to our knowledge, this is the first study to provide this information about the ST habits of endurance runners. Regarding this, athletes should take into account the potential relevance of heavy ST, given its potential for improving neuromuscular efficiency, delaying the activation of less efficient type II fibers or converting fast-twitch type IIx fibers into more fatigue-resistant type IIa fibers, and, in general, improving endurance performance [18]. The timing or moment in which ST sessions are performed might be other interesting characteristic for consideration. Current results indicate that most of the endurance athletes (i.e., 52.7%) included ST sessions on alternating days with running sessions, while 29.3% of the athletes combined different times for these ST sessions. Only the study by Blagrove et al. [16] considered the timing of the ST session, and the authors reported similar results compared to the results reported here, with ~50% of the athletes including RT in independent workouts.

A growing body of evidence points toward the importance of specific muscle groups (e.g., core [19], intrinsic foot muscles [20,21], and respiratory muscles [22]) and their training for both maximizing performance and minimizing risk of injury in endurance runners. Nevertheless, few studies have considered this topic. One of them [16] concluded that 14.8% of the athletes included barefoot exercises and 70.2% used core stability exercises in their training routines. Other work [17] reported that half of Irish runners (47.9%) trained core muscles twice a week. The results reported are lower compared to the current work, with 90.1% of athletes from this study including core training and 38.2% including foot muscles training. Additionally, as a novelty, this study indicates that 24.2% of athletes included respiratory muscles training.

A further finding of the current study is related to the influence of athletic level. A previous study considered the potential influence of athletic level on the training characteristics of elite and national class endurance runners [15], concluding that higher-level runners completed more ST than lower-level runners. In line with this study, our research revealed significant differences between level groups, with higher-level athletes including more S&C activities in their training programs.

In order to control the potential influence of sex on the comparison between group levels, an independent analysis was conducted for each sex, establishing group levels for male and female endurance runners independently. Whereas some similar previous studies included male and female athletes [15,16,17], only the study carried out by Karp [15] examined the between-sex differences in the training characteristics of high-level endurance runners. That study included 104 male and 151 female endurance runners and reported some differences in the training characteristics (e.g., weekly mileage or periodization model) but it excluded S&C habits. The current study is not focused on sex comparisons, but it provides an independent analysis in order to properly examine the influence of athletic level. The findings reported here are similar for both sexes, with S&C habits being influenced by the level of the runners.

Finally, some limitations need to be addressed. First, there was a lack of sex comparisons [23]. In the current study, a group of 228 women participated, but the authors decided to perform an independent analysis for each sex and not to incorporate sex comparisons, given that most of the female athletes were in the LG1 and LG2 (i.e., lower-level groups) when the group were considered as a whole. Since the athletic level seems to be an influencing factor on the S&C habits, a comparison with the group of men would have implied a bias. Secondly, there was a lack of information about running-related injuries. The inclusion of some items about prevalence and incidence of injuries would allow the authors to discuss the role of ST in an injury prevention context. Notwithstanding these limitations, the current study examines the S&C habits of 1179 Spanish amateur endurance runners while also considering the influence of athletic level.

## 5. Conclusions

Comparing the ST habits between endurance runners of different athletic levels, our results highlight that the frequency of ST increased as the athletic level increased. That is, higher-level runners included more types of ST and did ST more frequently than lower-level athletes, regardless of sex.

Most of the endurance runners included some type of ST into their training programs, with uphill runs, RT, and bodyweight exercises being the most common activities, whereas less than half of the runners included plyometric training into their weekly plans. Regarding the frequency, most of the runners performed ST twice a week, usually with low-to-moderate loads and a high number of repetitions. Ultimately, the between-level comparison suggests that high-level athletes selected the most effective S&C activities reported by scientific evidence (i.e., ST, RT, plyometric training, and resisted runs, and complementary training of core, respiratory, and foot muscles).

From a practical standpoint, this information might be of interest not only for coaches, to improve the inclusion of ST in the training program of endurance runners, but also for athletes, to understand the relevance of heavy ST, plyometric training, and resisted runs, beyond the traditional criterion of weekly training volume.

## Figures and Tables

**Table 1:** Frequency and prevalence (*n*, %) of S&C activities for male endurance runners regarding athletic level.

Variables	Strength and Conditioning	LG (*n* = 72)	LG2 (*n* = 126)	LG3 (*n* = 237)	LG4 (*n* = 324)	LG5 (*n* = 192)	*p*-Value
Strength and conditioning activities	No ST	14 (19.4) ^b,c,d,e^	18 (14.3) ^a,d,e^	30 (12.7) ^a,d,e^	21 (6.5) ^a,b,c,e^	6 (3.1) ^a,b,c,d^	<0.001
	RT	41 (56.9) ^d,e^	66 (52.3) ^c,d,e^	147 (62.1) ^b,d,e^	219 (67.5) ^a,b,c,e^	140 (73.0) ^a,b,c,d^	
	BW	36 (50.0) ^b,c,d,e^	89 (70.6) ^a^	157 (66.2) ^a^	223 (68.9) ^a^	140 (72.9) ^a^	
	PT	20 (27.7) ^b,c,d,e^	60 (47.6) ^a,c,d,e^	94 (39.7) ^a,b,d,e^	173 (53.3) ^a,b,c,e^	112 (58.3) ^a,b,c,d^	
	Uphill runs	33 (45.8) ^b,c,d,e^	81 (64.2) ^a,d^	157 (66.2) ^a,d^	233 (71.9) ^a,b^	129 (67.2) ^a^	
	Resisted runs	12 (16.7) ^c,e^	20 (15.8) ^c,e^	25 (10.5) ^a,b,e^	43 (13.2) ^e^	63 (32.8) ^a,b,c,d^	
	Combination	44 (61.1) ^b,c,d,e^	98 (77.8) ^a,c,d^	173 (72.9) ^a,b,d,e^	158 (48.8) ^a,b,c,e^	152 (79.2) ^a,c,d^	
Core	Yes	60 (83.3) ^b,c,d,e^	112 (88.9) ^a,e^	214 (90.3) ^a,e^	293 (90.4) ^a,e^	181 (94.3) ^a,b,c,d^	0.061
	No	12 (16.7)	14 (11.1)	23 (9.7)	31 (9.6)	11 (5.7)	
Intrinsic foot muscles	Yes	17 (23.7) ^b,c,d,e^	41 (32.5) ^a,c,d,e^	64 (27.2) ^a,b,d,e^	148 (45.6) ^a,b,c,e^	80 (41.8) ^a,b,c,d^	<0.001
	No	55 (76.3)	85 (67.5)	173 (72.8)	176 (54.4)	112 (58.2)	
Respiratory muscles	Yes	7 (9.8) ^b,c,d,e^	25 (19.8) ^a,c,d,e^	37 (15.7) ^a,b,d,e^	82 (25.4) ^a,b,c,e^	66 (34.3) ^a,b,c,d^	<0.001
	No	65 (90.2)	101 (80.2)	200 (84.3)	242 (74.6)	126 (65.7)	

Percentages are calculated relative to the number of runners per level of group. a, b, c, d, and e: denotes significant differences (*p* < 0.05) compared to LG1, LG2, LG3, LG4, and LG5, respectively. ST: strength training; RT: resistance training; BW: bodyweight exercises; PT: plyometric training; LG1, LG2, LG3, LG4, and LG5: denotes groups of athletes that ran a 10-km trial in 50–55 min, 45–50 min, 40–45, 35–40 min, and 30–35 min, respectively.

**Table 2:** Frequency and prevalence (*n*, %) of S&C activities for female endurance runners regarding athletic level.

Variables	Strength and Conditioning	LG1 (*n* = 60)	LG2 (*n* = 69)	LG3 (*n* = 72)	LG4 (*n* = 18)	LG5 (*n* = 9)	*p*-Value
Strength and conditioning activities	No ST	12 (20) ^b,c,d,e^	7 (10.1) ^a,c^	3 (4.2) ^a,b,d,e^	2 (11.1) ^a,c^	1 (11.1) ^a,c^	0.010
	RT	24 (40) ^b,c,d,e^	35 (50.7) ^a,d,e^	44 (61.1) ^a,d,e^	12 (66.6) ^a,b,c,e^	7 (77.8) ^a,b,c,d^	
	BW	20 (33.3) ^b,c,d,e^	33 (47.8) ^a,c,d,e^	39 (54.2) ^a,b,d,e^	11 (61.1) ^a,b,c,e^	6 (66.7) ^a,b,c,d^	
	PT	20 (33.3) ^b,c,d,e^	28 (40.6) ^a,c,d,e^	35 (48.6) ^a,b,d,e^	6 (55.5) ^a,b,c,e^	8 (88.9) ^a,b,c,d^	
	Uphill runs	23 (38.3) ^b,c,d,e^	36 (52.2) ^a,c,d,e^	39 (54.2) ^a,b,e^	12 (66.7) ^a,b,e^	7 (77.8) ^a,b,c,d^	
	Resisted runs	0 (0) ^c,d,e^	3 (4.3) ^c,d,e^	5 (6.9) ^a,d,e^	5 (27.8) ^a,b,c,e^	5 (55.6) ^a,b,c,d^	
	Combination	24 (40) ^b,c,d,e^	41 (59.4) ^a,c,d,e^	49 (68.0) ^a,b,e^	13 (72.2) ^a,b,e^	8 (88.9) ^a,b,c,d^	
Core	Yes	50 (83.3) ^b,c,d,e^	61 (88.4) ^a,c,d,e^	69 (95.8) ^a,b,d,e^	17 (94.4) ^a,b,c,e^	8 (88.9) ^a,b,c,d^	0.004
	No	10 (16.5)	8 (11.6)	3 (4.2)	1 (5.6)	1 (11.1)	
Intrinsic foot muscles	Yes	20 (33.3)	21 (30.4)	21 (29.2)	9 (50)	6 (66.7)	0.837
	No	40 (66.7)	48 (69.6)	51 (70.8)	9 (50)	3 (33.3)	
Respiratory muscles	Yes	8 (13.3)	13 (18.8)	20 (27.8)	9 (50)	7 (77.7)	0.200
	No	52 (86.7)	56 (81.2)	52 (72.2)	9 (50)	2 (22.3)	

Percentages are calculated relative to the number of runners per level group. a, b, c, d, and e: denotes significant differences (*p* < 0.05) compared to LG1, LG2, LG3, LG4, and LG5, respectively. ST: strength training; RT: resistance training; BW: bodyweight exercises; PT: plyometric training; LG1, LG2, LG3, LG4, and LG5: denotes groups of athletes that ran a 10-km trial in 50–55 min, 45–50 min, 40–45, 35–40 min, and 30–35 min, respectively.
